# The effectiveness and applicability of different lifestyle interventions for enhancing wellbeing: the study design for a randomized controlled trial for persons with metabolic syndrome risk factors and psychological distress

**DOI:** 10.1186/1471-2458-14-310

**Published:** 2014-04-04

**Authors:** Raimo Lappalainen, Essi Sairanen, Elina Järvelä, Sanni Rantala, Riitta Korpela, Sampsa Puttonen, Urho M Kujala, Tero Myllymäki, Katri Peuhkuri, Elina Mattila, Kirsikka Kaipainen, Aino Ahtinen, Leila Karhunen, Jussi Pihlajamäki, Heli Järnefelt, Jaana Laitinen, Eija Kutinlahti, Osmo Saarelma, Miikka Ermes, Marjukka Kolehmainen

**Affiliations:** 1 Department of Psychology, University of Jyväskylä, Ylistönmäentie 33, P. O. Box 35, 40014 Jyväskylä, Finland; 2 Institute of Public Health and Clinical Nutrition, Clinical Nutrition, University of Eastern Finland, Kuopio, Finland; 3 Institute of Biomedicine, University of Helsinki, Helsinki, Finland; 4 Finnish Institute of Occupational Health, Helsinki, Finland; 5 Department of Health Sciences, University of Jyväskylä, Jyväskylä, Finland; 6 VTT Technical Research Centre of Finland, Tampere, Finland; 7 Duodecim Medical Publications Ltd, Helsinki, Finland

**Keywords:** Lifestyle, Well-being, Obesity, Stress, Acceptance and Commitment Therapy, Cognitive behavioral therapy, Mobile application, Web-based intervention, Technology-aided interventions

## Abstract

**Background:**

Obesity and stress are among the most common lifestyle-related health problems. Most of the current disease prevention and management models are not satisfactorily cost-effective and hardly reach those who need them the most. Therefore, novel evidence-based controlled interventions are necessary to evaluate models for prevention and treatment based on self-management. This randomized controlled trial examines the effectiveness, applicability, and acceptability of different lifestyle interventions with individuals having symptoms of metabolic syndrome and psychological distress. The offered interventions are based on cognitive behavioral approaches, and are designed for enhancing general well-being and supporting personalized lifestyle changes.

**Methods/Design:**

339 obese individuals reporting stress symptoms were recruited and randomized to either (1) a minimal contact web-guided Cognitive Behavioral Therapy-based (CBT) intervention including an approach of health assessment and coaching methods, (2) a mobile-guided intervention comprising of mindfulness, acceptance and value-based exercises, (3) a face-to-face group intervention using mindfulness, acceptance and value-based approach, or (4) a control group. The participants were measured three times during the study (pre = week 0, post = week 10, and follow-up = week 36). Psychological well-being, lifestyles and habits, eating behaviors, and user experiences were measured using online surveys. Laboratory measurements for physical well-being and general health were performed including e.g. liver function, thyroid glands, kidney function, blood lipids and glucose levels and body composition analysis. In addition, a 3-day ambulatory heart rate and 7-day movement data were collected for analyzing stress, recovery, physical activity, and sleep patterns. Food intake data were collected with a 48 -hour diet recall interview via telephone. Differences in the effects of the interventions would be examined using multiple-group modeling techniques, and effect-size calculations.

**Discussion:**

This study will provide additional knowledge about the effects of three low intensity interventions for improving general well-being among individuals with obesity and stress symptoms. The study will show effects of two technology guided self-help interventions as well as effect of an acceptance and value–based brief group intervention. Those who might benefit from the aforesaid interventions will increase knowledge base to better understand what mechanisms facilitate effects of the interventions.

**Trial registration:**

Current Clinical Trials NCT01738256, Registered 17 August, 2012.

## Background

Obesity is increasing globally and is correlated with a variety of health problems. Consequences of being overweight might result in the metabolic syndrome, which is a set of medical features such as impaired glucose metabolism, dyslipidaemia and hypertension that are risk factors for cardiovascular disease and type-II diabetes [1,2]. Lifestyle changes have proven to be an efficient way to counter obesity and related disorders [3,4], however it is challenging to achieve long-term lifestyle changes using limited resources in real-life health care systems.

Individuals having problems related to overweight also experience additional health issues such as stress. Psychological stress is known to be related with increased body mass. In addition psychological stress also contributes to weight gain [5-7]. The fourth European Survey of Working Conditions reported that 22% of working European individuals suffers from stress. It is important to note that the annual financial cost for work-related stress within the EU was estimated at €20,000 million [8].

Much of existing health service models have problems in supporting long-term lifestyle changes required for managing of weight and stress. In addition, existing disease prevention and management models are not cost effective and do not necessarily reach those individuals who need them the most. A large number of studies indicate that lifestyle changes could be promoted by using different types of Internet-based technologies [9-14]. Available literature provides significant evidence that supports the effectiveness of web-based interventions of lifestyle and behavioral changes [15,16], however what type/s of web-based interventions could be beneficial for individuals with chronic health disorders is vaguely reported and need further investigation. In addition, little is known about the relevance of low intensity programs using novel interactive technologies offering minimal contact for those reporting both obesity and stress.

Additional controlled studies are necessary to evaluate different types of low intensity intervention models for prevention and treatments based on self-management. There are several options to facilitate development of low intensity self-management approaches for lifestyle-related chronic conditions, for example, use of Internet-administrated assessment, online health screening and coaching approaches based on current knowledge of Cognitive Behavioral Therapies (CBT). Low intensity CBT-based interventions use a minimum level of intervention essential to enhance mental health and well-being [17]. Much of the low intensity interventions use novel interactive communication technologies. They might support long-term lifestyle changes because they deliver swift and easy access to early intervention and preventive programs.

In addition to investigating the effect of low intensity web-based health screening and coaching application, we wanted to explore the effect of third wave CBT methods by applying mindfulness and acceptance-based interventions for individuals with obesity and stress. There has been an increasing interest towards these novel approaches lately with an aim to improve psychological flexibility for promoting life style changes. It has been proposed that psychological flexibility is one of the key requisites for psychological health [18]. Psychological flexibility is reflected by how a person: adapts to inconsistent situations, reconfigures mental resources, modifies viewpoints, and balances competing desires and needs. For example, interventions that include components of psychological flexibility have demonstrated significant benefits when compared with controlled conditions on measures of depression and anxiety [19]. Additionally, there is evidence suggesting that lower psychological flexibility and burnout increase the risk of unstable emotional state of mind and adverse eating habits [20]. These results among others indicate that improvement of psychological flexibility is undoubtedly beneficial for individuals suffering from obesity and/or stress.

Mindfulness, acceptance and value-based methods targeting psychological flexibility include psychological interventions such as Acceptance and Commitment Therapy (ACT) [21]. There are few studies that have investigated the effect of novel mindfulness and acceptance-based interventions such as ACT for chronic health conditions [22,23]. An increasing number of studies indicate that ACT is conceivably effective for a wide range of psychological and health disorder, such as chronic pain, smoking, diabetes, epilepsy and work-related stress [24,25]. Yet further studies are desirable to prove the effectiveness of ACT-based interventions such as mindfulness and acceptance practices in improving deteriorated metabolic indicators, such as dyslipidemia, low-grade inflammation or deficiencies in glucose metabolism.

ACT has mainly been investigated using face-to-face interventions, and there are only few studies that have studied the effect of ACT-based approaches for health disorders using web or mobile-based interventions. Web-based ACT interventions have been developed e.g. for tinnitus distress [26], chronic pain [27], and psychological stress [28]. Furthermore, ACT-based mobile apps have been studied on small scale [29,30]. To the best of our knowledge, there are very few controlled trials that have been conducted to investigate the effectiveness of ACT-based mobile interventions [29]. Overall, there are a limited number of controlled studies that have investigated the effects and feasibility of smartphone solutions for chronic health disorder [31].

### Aim and main hypotheses

The overall aim of the present study was to investigate the effect of three novel low intensity psychological interventions for metabolic syndrome risk factors, psychological flexibility and general well-being among obese individuals experiencing stress. The purpose was to study (a) the effects of minimal contact web-based CBT intervention including an approach of health assessment and coaching methods (Internet), (b) the effects of mobile-based intervention containing mindfulness, acceptance and value-based exercises (Mobile), and (c) the effects of face-to-face group intervention using mindfulness, acceptance and value-based approaches (Face-to-Face) in a controlled study design, where control (Control) received no treatment, but the same measurements were completed for all the groups. Thus, both technology-based low intensity self-help interventions offered minimal contact, and they were compared to a brief group intervention. Moreover, we were interested in studying acceptability, applicability, perceived benefits, user experiences, and usage of the said interventions.

The three interventions groups, and the control group were compared based on changes in lifestyle measures (e.g. food and nutrition intake, meal patterns, physical activity, sleeping habits), psychological measures (e.g. psychological flexibility, perceived stress, depression symptoms, quality of life, perceived quality and quantity of sleep), clinical and biochemical variables (e.g. BMI, body composition, circulating glucose, insulin, lipids, stress and inflammation indicators), other physiological measurements (e.g. objective sleep measurements, heart rate variability-based recovery from stress), and user experiences (e.g. experiences with the interventions, actual usage, realization of exercises presented in interventions). We formulized the following hypotheses:

H1: Participants who receive any of the three interventions would show improvements in lifestyle and psychological measures, and clinical, biochemical, and physiological variables as compared to the control group. Each intervention group is compared with the control condition separately.

H2: Participants who receive any of the three interventions will show equal positive changes in lifestyle, and psychological measures, and clinical, biochemical, and physiological variables when the interventions are compared with each other.

We also wanted to examine demographics and psychological variables that could predict change over time (Pre, Post and Follow-up) including age, gender, initial BMI, physical activity, psychological flexibility, stress and depressive mood and psychological symptoms. Further, we would examine potential mediators on the effect of the interventions including psychological flexibility and mindfulness skills. Secondary objectives include analyses of the user experiences and validity of used measurement and intervention methods.

## Methods/Design

### Study design

**Figure 1:**
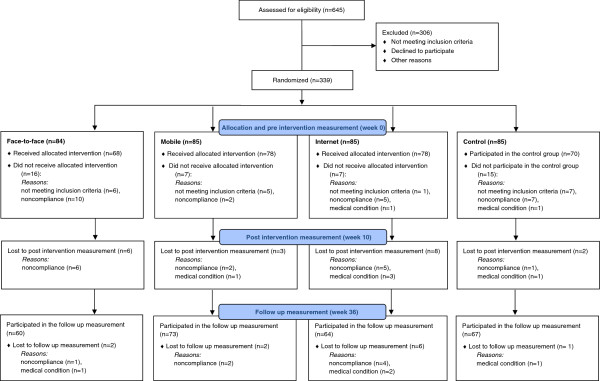
Flow chart of the study design.

This study was a randomized controlled trial (RCT) (Figure 1). Main outcome measures were psychological flexibility, weight, diet quality, eating behavior, perceived stress, depression symptoms, and sleeping habits. Several other variables associated with lifestyle changes, psychological and physiological health was assessed during the study. We were also interested in user experiences of the three novel interventions. The interventions are described below in detail.

The study was performed at three cities in Finland (Helsinki, Jyväskylä, and Kuopio) by the local universities (University of Helsinki, University of Jyväskylä, and University of Eastern Finland, Kuopio). In addition, VTT Technical Research Center of Finland (Tampere, Finland), Finnish Institute of Occupational Health (Helsinki and Oulu), and companies Duodecim Medical Publications Ltd. (Helsinki, Finland), Firstbeat Technologies Ltd. (Jyväskylä, Finland), Vivago Ltd. (Espoo, Finland), Finnish Red Cross Blood Service (Helsinki, Finland), and Valio Ltd. (Helsinki, Finland) participated in performing the study. The study was approved by the ethics committee of the Central Finland Health Care District, and registered with ClinicalTrials.gov with the identifier NCT01738256.

The study was carried out in two phases. The first phase started in September 2012 and the second phase in January 2013. The study included pre-measurements before the 8-week interventions, post-measurements 10 weeks after the pre-measurements, and follow-up measurements six months after the post measurements (36 weeks from pre-measurement). The last measurements were collected in December 2013.

### Study population

The recruited participants (n = 339, Figure 1) were either overweight or obese and reported psychological stress symptoms. The inclusion criteria were: 1) Body Mass Index (BMI) 27–34.9 kg/m^2^, 2) perceived psychological stress (at least 3/12 points in General Health Questionnaire [32], and 3) the possibility to use computer with internet connection. The exclusion criteria were: diagnosed severe chronic illness (symptomatic cardiovascular disease, Type I or II diabetes, severe psychiatric conditions or substance abuse), medical surgery within the past 6 months, heart attack/stroke within past 6 months, kidney disease requiring dialysis or other disabilities/illnesses affecting substantially physiological or mental health, regular use of cortisone pills, pacemaker, eating disorder (bulimia), disability pension for psychological reasons, pregnancy or breastfeeding within the past 6 months, shift work (in three shifts) or night work, psychotherapy or other psychological or mental treatment at least twice a month, and participation in other intervention studies during the present study.

### Procedure for recruitment, randomization and allocation

The participants were recruited by using advertisements in local newspapers. All the participants took part in the study on volunteer basis. The initial exclusion criteria were assessed through phone calls and by using electronic questionnaire for reported distress. The participants who passed the initial screening were randomly allocated into one of the three intervention groups (Internet, Mobile, Face-to-Face), or into a control group. After randomization, participants with predetermined abnormalities in baseline laboratory examinations were excluded from the study (see Figure 1). At the baseline visit to the study center the blood samples were drawn for determining health status and excluding volunteers with unknown/undiagnosed chronic disease or other health problem. The measurements for liver, thyroid glands and kidney function as well as glucose and lipid metabolism were taken. If there was a value outside reference values, the participant was excluded before starting the actual interventions. Written informed consent was obtained during the laboratory examination. Randomization was done by a university statistician using a table of random numbers (in batches of four). An independent person outside of the research group prepared envelopes containing the randomization number, study ID and group number by center. The envelopes were opened in the order participants passed the initial screening following ‘concealment of allocation’ principle.

### Interventions

#### Internet-based coaching

The Internet-based intervention (Table 1) consisted of a 12-week program without any face-to-face contact (Duodecim Virtual Health Check and Coaching program). The intervention combined the assessment of psychological resources and health related behavior to provide users with a comprehensive view of their health and life situation and ways to improve it. With the support of the assessment, the users could choose between coaching programs to improve positive life skills and to change health related behaviors.

**Table 1:** Content of the Internet intervention

**Content**	**Key points**
Health questionnaire	Estimate of life expectancy, coronary heart disease, stroke and diabetes risk. Description of one’s life habits and behaviors impacting on health and ways to influence them
**Coaching programs**	
Each program includes a weekly message.	
In weight management, healthy diet, exercise, sleep improvement and alcohol use management programs the user has an option to record follow-up information about relevant behavior or parameter:	
a) Weight management	Messages consist of information, practical advice and exercises on weight management (e.g. managing appetite, eating, portion size, buying food), and link to further readings.
b) Healthy diet	Messages about healthy diet and practical advice for improvement, and also links to further readings.
c) Exercise	Messages include information about health related physical activity and link to further readings. Message contains also advice or practice.
d) Stress and life management	Optional: Stress management, Good deeds, Optimism, Human relations, Social relations, Positive interaction in relationships, Resolving conflicts in relationships, Coaching exercises for families with children. The weekly coaching message includes information, practical advice, and exercise about stress or life management and link to further readings.
Optional:	
e) Sleep improvement	Information, practical advice and exercises on good sleep (e.g. sleep hygiene, environment, relaxation) and link to further readings. Message contains also advice or practice.
f) Alcohol use management	Cognitive behavioral program to analyze reasons and situations of alcohol use and advice to avoid excess use.
g) Smoking cessation	Cognitive behavioral program to analyze reasons and situations of smoking, mental exercise, and support for quitting.

**Figure 2:**
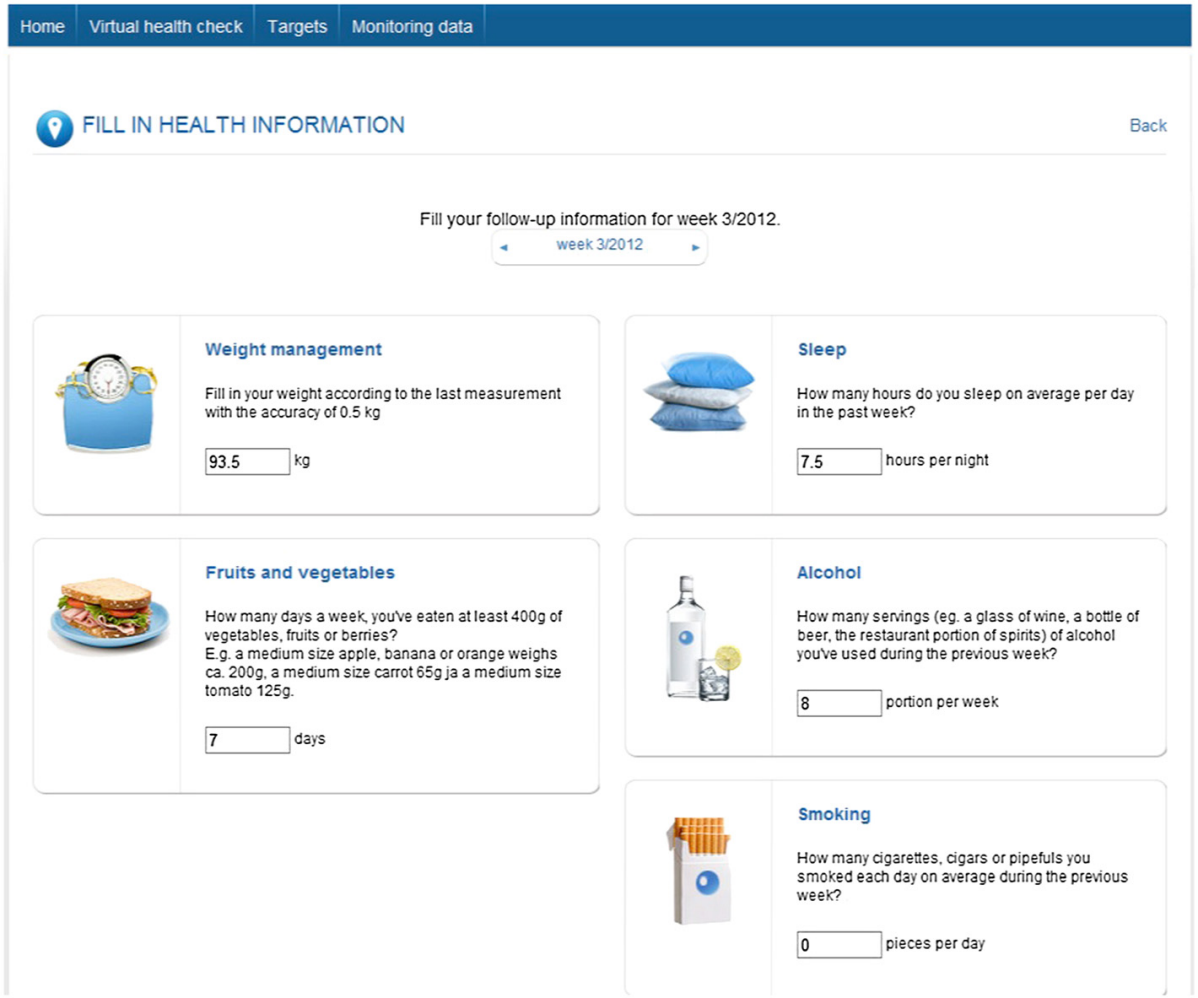
Screenshots of the internet application.

**Figure 3:**
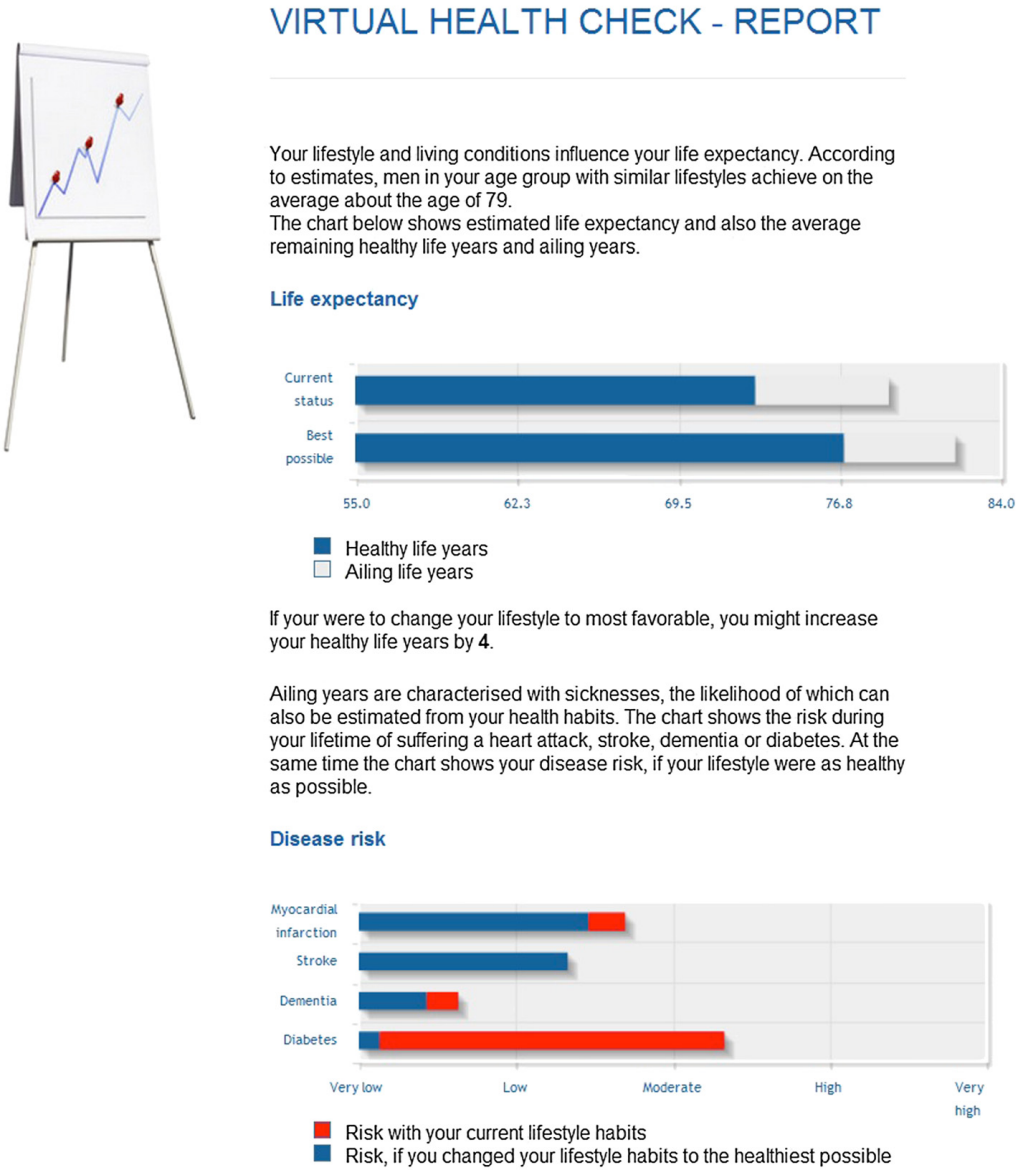
Screenshots of the internet application.

In the Health Check, the users filled out a health questionnaire assessing key health determinants, lifestyle factors, emotional well-being, and performance (Figure 2). On the basis of the assessment, the users received estimations, including a prognosis of average life expectancy, coronary heart disease, stroke and diabetes risk, as well as a description of their habits and behaviors impacting their health (Figure 3). The questionnaire and algorithms assessing health risks were based on the Finnish health check and follow-up study Finriski, having up to 30 years follow-up data on a population sample more than 10 000 people [33].

On the basis of the health check feedback, users could choose optional coaching programs for weight management, healthy diet, exercise, sleep improvement, alcohol consumption management, smoking cessation, and mental well-being (stress management, gratitude, forgiveness, good actions, optimism, positive interaction in relationships and resolving conflicts in relationship). Participants were nonetheless commended to choose primarily from the following three programs: Stress and life management, Nutrition, and Exercise. The programs were designed to support improvements in the health and well-being habits and behaviors.

**Figure 4:**
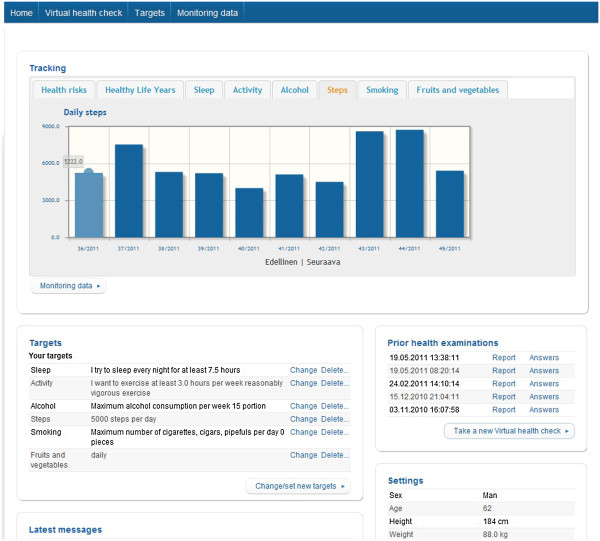
Screenshots of the internet application.

In the coaching program, users received a weekly coaching message including information about the chosen topic and a link to further information. In addition, the message contained advice or exercise, through which an individual could improve awareness of his/her behavior and practice skills to develop habits that support wellbeing. The weekly message also included feedback based on entries made by the respective user. The users had an option to record follow-up information about their lifestyles (weight, physical activity, nutrition, alcohol consumption and smoking). During the program, users could monitor their progress through graphs, make new health checks, and choose new coaching programs (Figure 4).

Coaching programs for weight management, sleep improvement, exercise, alcohol consumption management, and smoking cessation were based on cognitive-behavioral practices on lifestyle changes [34-37]. The test and coaching programs for mental well-being have been developed along the outlines described by several authors [38-40] showing that the cognitive behavioral coaching program to develop optimism, gratitude and other life skills are effective in traditional writing exercise and web-based coaching programs [38,41]. The validity of the variables has been tested in a large cross-sectional study where more than 130 000 Finns completed the questionnaire [42].

### Mobile-based intervention

The participants in the Mobile-based intervention were invited in a group meeting that consisted of a brief overview of Acceptance and Commitment Therapy (ACT) principles. In the meeting, the participants were given Android smartphones that were pre-installed with a stand-alone mental wellness training application called Oiva. They were instructed to use Oiva for the upcoming 8 weeks at their own. The application contained short exercises that taught ACT skills to be applied in daily life. The mobile application delivered an ACT-based intervention program similar to the Face-to-Face group. Prior to the present trial, the application had been piloted in a feasibility study with a sample of office workers with stress symptoms [29].

The content of the application was divided into four intervention paths: Mindful Mind, Wise Mind, Values, and Healthy Body (Table 2). The first three paths demonstrate core processes of ACT and the fourth path applies ACT-based approach on physical well-being. The application contained altogether 46 exercises in text and audio formats, as well as introduction videos for each path and section. The program did not include psycho-education on healthy diet or physical activity, only a hyperlink to a public nutritional website was provided.

**Table 2:** Content of the four paths in the mobile intervention

**Topic of the path**	**Content**	**Key points**
Mindful mind	Five mindfulness exercises	Contact with the present moment.
		Focusing fully on one’s inner or outer experiences.
		Acting mindfully.
Wise mind	Observation: six exercises	Observing thoughts without being caught up in them.
	Acceptance: four exercises	Making room for unpleasant feelings, sensations, urges, and other private experiences; allowing them to come and go without struggling with them.
Values	Values: seven exercises	How I use my time in my current life?
		How mindfulness-skills can improve wellbeing?
		What are the most important values for me?
		Am I living according to my values?
	Value based actions: seven exercises	What are my specific goals and actions that support my valued behavior?
Healthy body	Relaxation: six exercises	Relaxing and listening to my body.
	Mindful eating: six exercises	Exercising mindful eating. What is my typical meal rhythm?
	Physical activity: five exercises	Everyday physical activity in small steps.
	Eleven gymnastic video clips	

**Figure 5:**
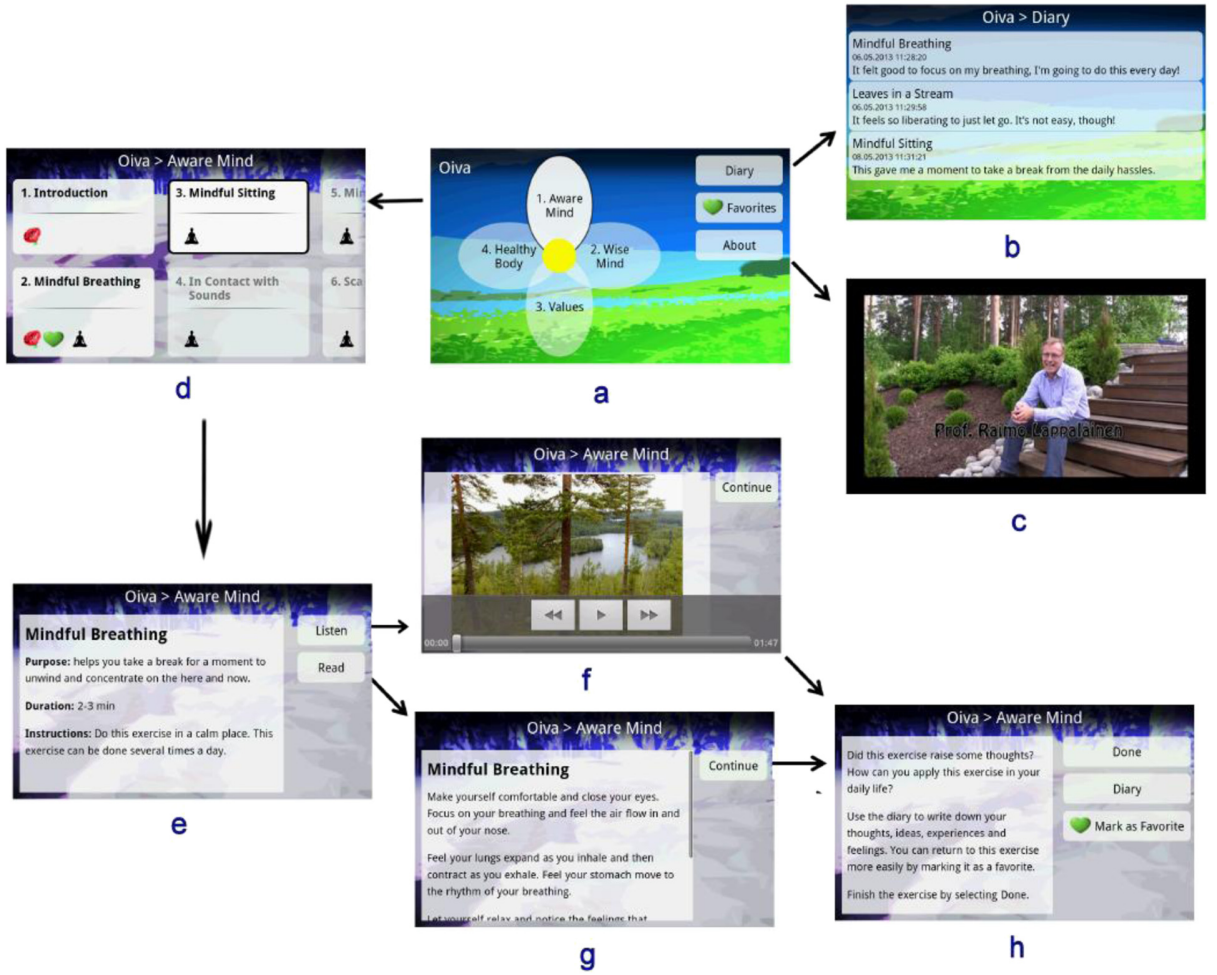
**Screenshots of the mobile application. (a)** main screen, **(b)** diary, **(c)** introduction video, **(d)** top menu of Mindful Mind, **(e)** exercise introduction screen, **(f)** audio exercise, **(g)** text exercise, **(h)** exercise reflection screen.

Figure 5 presents examples of the user interface of the application. The main screen (Figure 5a) contained a flower-shaped menu through which different paths could be accessed. The main screen also provided access to a diary (Figure 5b), list of favorite exercises, and introduction to the application in text and video formats (Figure 5c). Each petal represents one of the paths, which were numbered according to their recommended order. Each path consisted of 1–4 subsections (“steps”), which included 5–8 exercises (Figure 5d). There was an introduction in text and video formats to each path and step, informing users about the processes and skills.

Most of the exercises were short and took about 1–3 minutes to be completed (Figure 5e-h). This aimed at making exercises easy to perform in any situation. Each exercise began with an introduction presenting the purpose, duration and instructions of the exercise (Figure 5e). The users could choose to exercise by listening (Figure 5f) or by reading (Figure 5g). After each exercise, a reflection screen (Figure 5h) summarized the skills learned from the exercise and enabled users to write notes and reflections in their diary (Figure 5b). The notes were saved in the diary and could be accessed later. The application smoothly guided users through the intervention program without restricting free navigation. Paths, steps, and exercises were numbered in the recommended order and the next suggested item was dynamically highlighted (Figure 5a and d). However, all paths and exercises were accessible from the very beginning.

### Face-to-face group intervention (Face-to-Face)

The ACT-based face-to-face group intervention consisted of six group sessions during 8-week period of time, with each session lasting for about 90 minutes (see Table 3). Each group consisted of 6–12 participants and was instructed by a psychologist. Three psychologists acted as coaches. All of them were trained in ACT approach, had experience in ACT interventions, and had a manual of the program to follow. The participants were given a workbook that contained short descriptions of the sessions, exercises, and individual reflections and notes.

**Table 3:** Content of the six group sessions in the Face-to-Face group intervention

**Topic of the group session**	**Content**	**Key point of the session**
My life here and now	Values: one exercise	How I use my time in my current life?
	Mindfulness: one exercise	Contact with the present moment.
Values and mindful living	Values: three exercises	How mindfulness-skills can improve wellbeing?
	Mindfulness: one exercise	What are the most important values for me?
	Mindful eating: one exercise	Am I living according to my values?
Value-based actions and barriers	Action: one exercise	What are my specific goals and actions that support my valued behavior?
	Mindfulness: one exercise	My subjective barriers or reasons connected to healthy eating or physical activity.
	Observation: three exercises	
The observing self and acceptance	Observation: one exercise	Observing thoughts without being caught up in them (defusion).
	Acceptance: three exercises	Making room for unpleasant feelings, sensations, urges, and other private experiences; allowing them to come and go without struggling with them.
	Relaxation: one exercise	Can you accept yourself as you are?
Mindful eating	Mindfulness: one exercise	Exercising mindful eating.
	Mindful eating: three exercises	What is my typical meal rhythm?
Summary and reflection	Observation: one exercise	What have I learned?
	Values: one exercise	How to continue with lifestyle changes?
	Acceptance: one exercise	

The intervention program aimed to support lifestyle changes and to enhance well-being through committed and concrete actions based on the personally important values – the most important directions in life. The program started with an analysis of own life situation and reflections about values and important things in life. After the value clarification, the participants made concrete action plans and defined their own goals. The emphasis was placed on small and feasible actions that lead to better life according to personally chosen values. In order to achieve the set goals, the participants were taught acceptance and mindfulness skills and new ways to deal with the barriers of action. The purpose of teaching acceptance and mindfulness skills was to increase the ability to become aware of one’s own thoughts, emotions and instinctive behavior patterns. By noticing these instinctive thinking styles or routines, and by producing greater psychological flexibility, the participants were expected to have better skills to consciously change their behaviors in everyday life.

Every session included mindfulness exercises, pair and group discussions, and homework related to the topic of the session. The intent behind homework was to encourage participants to work with the topics between the sessions and apply learnt skills into their own living context. The group leader’s role was to introduce the topics, teach new skills, lead the on-going discussions and provide a context in which participants are willing to share their own experiences, thoughts, and feelings. The program did not include psycho-education on healthy diet or physical activity, only a hyperlink to a public nutritional website was provided.

### Control group

The participants randomized in the control group participated in all the measurements but were not part of any active intervention.

### Measurements

All the participants were measured at the beginning of the study (pre-measurement), after 10 weeks (post-measurement), and after 36 weeks (follow-up). The study included a wide range of psychological, physiological, and technological measurements. The main variables are described below in more detail.

### Psychological measurements using questionnaires

#### ***Perceived stress***

The Perceived Stress Scale (PSS) evaluated participants’ subjective experience of stress. PSS was developed to measure the degree to which situations in one’s life are appraised as stressful [43]. PSS is a widely used method that assesses nonspecific perceived stress.

#### ***Depression***

The Beck Depression Inventory-II [44] was used to measure symptoms of depression. It is one the most commonly used tools in research and practice to measure the presence and severity of depression. Depressive symptoms can be categorized into no/minimal depression (0–13 points), mild depression (14–19 points), moderate depression (20–28 points), and severe depression (29–63 points) levels.

#### ***Sense of coherence***

Sense of coherence (SOC-13) was measured by the 13-item Orientation to Life –Questionnaire [45,46]. The scale consists of three dimensions: Comprehensibility (five questions), Manageability (four questions), and Meaningfulness (four questions). The participants were asked to answer the questions on a seven-point semantic differential scale from 1 (= never) to 7 (= always) with the total sum ranging from 13 (low SOC) to 91 (high SOC).

#### ***Quality of life***

The RAND-36 [47,48] is widely used health-related quality of life instrument. It comprises of 36 items that assess eight health features: physical functioning, role limitations caused by physical health problems, role limitations caused by emotional problems, social functioning, emotional well-being, energy/fatigue, pain, and general health perceptions. Physical and mental health summary scores are also derived from the eight scales.

#### ***Psychological flexibility***

Psychological flexibility was assessed using the general Acceptance and Action Questionnaire (AAQ-II) [49] and the Acceptance and Action Questionnaire for Weight (AAQW) [50]. The Acceptance and Action Questionnaire (AAQ-II) [49] is a 7-item Likert-type questionnaire that assesses the ability to accept aversive internal experiences and to pursue goals in the presence of these experiences. In previous research, it has been found that mediation of specific ACT protocol by ACT process is better assessed by modifying the general AAQ to target the specific area [22,51]. For that reason, the present study also used a targeted measure adapted from the original AAQ as a process measure. The Acceptance and Action Questionnaire for Weight [50] is a 22-item, Likert-type scale that measures acceptance of weight-related thoughts and feelings and the degree to which they interfere with valued action (e.g., “I try hard to avoid feeling bad about my weight or how I look”, [50].

#### ***Mindfulness skills***

Mindfulness skills were investigated using Five Facet Mindfulness Questionnaire (FFMQ). FFMQ [52] is based on a factor analytic study, and the analysis yielded five factors that represent elements of mindfulness as it is currently conceptualized. The five facets are observing, describing, acting with awareness, non-judging of inner experience, and non-reactivity to inner experience. The questionnaire includes 39 questions (for example, “I’m good at finding words to describe my feelings”, “I rush through activities without being really attentive to them”). Participants are asked rate each of the statements using the scale from never or very rarely true to very often or always true.

### Lifestyle measurements using questionnaires, interviews and diaries

#### ***Physical activity***

Leisure time and work-related physical activity as well as sedentary time was assessed by a questionnaire of present activity and changes within the last two months. The assessment of leisure time physical activity is based on a series of structured questions covering leisure-time physical activity and commuting activity allowing calculation of a validated sum score of leisure time physical activity volume [53,54].

#### ***Sleep***

Self-reported sleep and insomnia symptoms were assessed through the 7-day sleep diary [55] accompanied by actigraphy (see objective physiological measuring). The main symptoms for insomnia were assessed by the items about sleep onset latency (SOL), and wake after sleep onset (WASO). The timing of the sleep period was elicited by the questions concerning times of going to and getting out of bed, from which total time in bed (TIB), total sleep time [TIB - (SOL + WASO)] and sleep efficiency (TST/TIB X 100) were calculated. The sleep diary contained questions about timing of work, quality of sleep, daytime alertness, perceived stress, daily physical activity, and about the use of sleep promoting medication, other medications and alcohol during the day.

In addition, self-reported sleep and insomnia symptoms were assessed by 10 questions of the Basic Nordic Sleep Questionnaire (BNSQ) [56] about habitual sleep time sleep need, and sleep-related symptoms during the past month. Epworth sleepiness scale [57] was used to asses self-reported daytime sleepiness. Where participants rated the probabilities that they might fall asleep in eight situations commonly encountered in daily life.

#### ***Food and nutrient intake and meal pattern***

Food intake was assessed by a 48 hour dietary recall interview. Three trained nutritionists who were involved in the development of the interview protocol carried out interviews. Interviews were performed at a prearranged time by phone at each time point of the study (week 0, 10 and 36). The same nutritionist interviewed each participant. Interviews were carried out from Tuesday to Friday to ensure that interview days were not weekends. Participants were requested to have access to a computer during the interview in order to use an electronic picture book of food portion sizes [58] sent by the email prior the interview. During the 48 h dietary recall interview the participants were asked what they had eaten or drunk, including portion sizes and cooking methods, time and place during the day. The AivoDiet software (Aivo Ltd., Turku, Finland) was used for food coding and calculating the nutritional composition of consumed foods and beverages. Nutritional calculations are based on the Fineli^®^ Finnish Food Composition Database (National Institute for Health and Welfare, Nutrition Unit, Helsinki, Finland). In addition, meal patterns concerning previous month were asked by the Finnish diet quality questionnaire to assess diet quality (for the Index of Diet Quality, [59]).

#### ***Eating behavior measures***

Eating behaviors were assessed by questionnaires. The Intuitive Eating Scale (IES-1) [60] is a 21-item scale that measures the tendency to eat according to internal hunger and satiety cues. The Three-Factor Eating Questionnaire Revised 18-item version (TFEQ-R18) [61] measures cognitive restraint, uncontrolled eating and emotional eating. The ecSatter Inventory for Low-Income (ecSI/LI) [62,63] consists of 16 items that quantify eating competence. Eating competence [64] includes positive attitudes to eating and to food, food acceptance skills that enable increasing variety of foods used, regulation of food intake based on internal cues, and the ability to successfully manage food consumption. From the Health and Taste Attitude Scales (HTAS) [65] were used 6-item subscales “Using food as a reward”, and “Pleasure”.

#### ***Other lifestyle –related measures***

Use of alcohol and tobacco, stomach symptoms (IBS) and work ability (Work Ability Index, WAI, [66]) were measured by questionnaires. The WAI was used to assess the individual work ability. It covers the following dimensions: an individual's current work ability compared with his/her lifetime best, and work ability in relation to the demands of the job; the number of diagnosed illnesses or limiting conditions from which he/she suffers; estimated impairment due to diseases/illnesses or limiting conditions; the amount of sick-leave he/she has taken over the last year; and individual’s own prognosis of his/her work ability in two years' time.

### Clinical and biochemical measures

#### ***Body composition, weight, BMI***

Multi-frequency bioelectrical impedance analysis of body composition were performed at each time point using In Body 720-device (Mega Electronics, Kuopio, Finland) or Tanita BC 418 MA–device (Tanita, Japan). The device provides information of entire body and appendicular fat mass and lean soft tissue mass. Body weight was measured using the same calibrated electronic scale in the study centers throughout the study. Waist circumference was measured halfway between the lowest rib and the iliac crest. A height gauge was used for height measurement. The body mass index (BMI) was calculated based on height and weight information.

#### ***Blood samples***

Antecubital venous blood samples were taken at study week 0, 10 and 36 after a 12 h overnight fast. Fasting serum γ- glutamyl transferase (GT), alanine transferase (ALAT), alkaline phosphatase (AFOS), thyroid stimulating hormone (TSH), full blood count (FBC), and creatinine, and on weeks 0, 10 and 36 to analyze total cholesterol, LDL-cholesterol, HDL-cholesterol, triglycerides and plasma glucose were analyzed using the routine methodology used in the study centers (University of Jyväskylä, Department of Health Sciences, University of Helsinki, Department of Biomedicine and HUSLAB, University of Eastern Finland, Kuopio, Institute of Public Health and Clinical Nutrition and ISLAB).

Fasting serum insulin (chemiluminescent immunoassay (Advia Centaur Immunoassay System, Siemens Medical Solution Diagnostics,Tarrytown, NY, USA), serum highly sensitive C-reactive protein (hs-CRP) nephelometry (Siemens, Eschborn, Germany), serum interleukin-1 receptor antagonist (IL-1Ra) (Quantikine Elisa Kits; R&D Systems Inc., Minneapolis, USA), serum high molecular weight adiponectin (HMW-adiponectin) (Quantikine ELISA for Human HMW Adiponectin/Acrp Immunoassay, R&D Systems Inc., Minneapolis, USA), plasma cortisol (LIAISON Cortisol, chemiluminescent immunoassay, Diasorin, Italy), and plasma dehydroepiandrosterone sulphate (DHEA) (LIAISON DHEA-S chemiluminescent immunoassay, Diasorin, Italy) were measured centrally at the University of Eastern Finland, Kuopio, Institute of Public Health and Clinical Nutrition.

### Objective physiological monitoring

#### ***3-day beat-to-beat heart rate measurement***

The participants completed a 3-day (72 hours) measurement of ambulatory R-to-R intervals of the heart in real life settings. Firstbeat Bodyguard measurement device (Firstbeat Technologies Ltd., Jyväskylä, Finland) and ECG electrodes (Ambu Ltd., Ballerup, Denmark) were used for collecting the RR-interval data. The data was analyzed with Firstbeat Health software (Firstbeat Technologies Ltd., Jyväskylä, Finland) that utilizes both heart rate (HR) and its variability (HRV) in the analysis. The software computes traditional HRV variables and also variables describing physiological states (e.g. stress, recovery, and physical activity) based both on HR, HRV and respiratory variables. By analyzing HRV it is possible to assess autonomic modulation of the heart [67], and HRV measurement is often used as physiological indicator for stress and recovery.

#### ***7-day movement based actigraphy and activity measurement***

Participants wore a Vivago Well-being Watch (Vivago Ltd., Espoo, Finland) on their non-dominant wrist for seven consecutive days for the analysis of their sleep time, calorie expenditure, and activity. The device’s screen was covered with a sticker during the measurements to avoid self-monitoring during the study. The device has been found to produce similar results as traditional actigraphs in wake/sleep detection [68,69]. The agreement percentage between traditional polysomnography and Vivago device has been approximately 80% that is comparable to other actigraphs in wake-sleep detection [69].

### User experiences and usage of technology

Participants’ prior experience of using web and/or mobile-based wellness technologies was assessed at the beginning of the study. Two weeks after the beginning of the study participants completed an online questionnaire about their initial experiences related to interventions. The aim was to investigate factors that facilitate or hinder introduction and adoption of mobile and/or web-based interventions. The post measurements included online user experience questionnaires for all the intervention groups. The questionnaires for Mobile and Internet groups assessed usability, acceptance, perceived benefits, motivational factors and usage of mobile and web interventions in long-term use. The questionnaire for the Face-to-Face group investigated experiences of the intervention as a whole and assessed its most beneficial aspects. The post measurements also involved brief (15 minute) phone interviews that collected qualitative data that provides deeper insights into user experiences and perceived benefits. Objective technology usage data was extracted from log files generated by the mobile and web applications.

### Feedback for each group after the study

Comprehensive information gathered during the study concerning personal health results and their interpretation guidelines were provided to the participants after the follow-up measurements (36 weeks). The feedback included information on laboratory measurements (blood samples), body composition analyses, diet feedback, and sleep and lifestyle assessments based on actigraphs and heart rate measurements. In addition, an "Eat well - working-age nutrition instruction" booklet (Ravitsemusterapeuttien yhdistys ry 2013) was provided after the intervention. The booklet contained information on the implementation of a regular meal pattern and healthy foods selection with hands-on examples. The control group was offered an opportunity to participate in a face-to-face meeting after the study was completed. In the meeting, interventions used in the study were introduced. The control group was also offered an opportunity to start the Internet-intervention as well as instructions to obtain mobile-intervention to their personal phones (the application was freely available at Google Play Store at the time of meeting).

### Statistical analysis

Data will be analyzed using the IBM SPSS statistics version 19.0 or newer and Mplus statistical package 7.1 [70]. Analyses will be controlled for possible baseline differences between the groups. Differences in the stability of the outcome measures between the groups will be examined using multiple-group modeling techniques. Full information maximum likelihood (FIML) estimation under the assumption of data missing at random (MAR) will be used in analyzing incomplete data. As the normality assumption is violated, maximum likelihood with robust standard errors (MLR) will be used. The significance level of the study will be set at 0.05. Cohen’s *d* will be used to estimate the effect size, and to reflect the clinical significance of the interventions. A between group effect size of 0.2 is considered small, 0.5 medium, and 0.8 large [71]. A within group effect size of 0.5 is considered small, 0.8 medium, and 1.1 large.

Power calculations were performed in order to determine the sufficient sample size to detect the significant intervention effect at a significance level of α = 0.05. Analysis was conducted for one of the primary outcome measures, depression symptoms (BDI-II). On the bases of our earlier studies using similar brief intervention procedures [72] we expect that the Post-measurement difference between the intervention and control group to be approximately 10 scores. A sample size of 320 (80–85 per group) was expected be sufficient at the power level of 1.00.

## Discussion

### The aim of the study

This article demonstrates a research protocol for a randomized controlled trial for studying different well-being programs among individuals with metabolic syndrome risk factors and perceived stress. The effects of three low intensity psychological interventions were investigated in a randomized controlled study design. We expect that the effect of a minimal contact web-based health assessment and coaching methods, and the effect of mobile-based intervention containing mindfulness, acceptance, and value-based exercises would be equal to the effect of face-to-face group intervention using mindfulness, acceptance, and value-based approaches. Further, we expect that these low intensity interventions would show larger changes in psychological and physical measurements of health and well-being when compared with the no treatment control group.

Examining the effects of low intensity interventions with reasonable cost-effectiveness is essential when we are dealing with common health problems. Both obesity and stress related issues are among the major health concerns at the moment [8,73]. In addition to the fact that lifestyle related health problems are very common, the current health service models face several challenges. For example, it has been reported that only a minority of individuals who may benefit from effective health services aiming at lifestyle changes actually use them or seek professional help [74,75]. Further, Internet contains self-help web pages and programs dealing with health and chronic health problem, but relatively few of them are based on evidence-based interventions. Thus, evidence-based low intensity interventions to which individuals have 24/7 access are feasible option to solve or prevent these problems [17]. Also, the development of interactive technologies expands opportunities to provide low intensity interventions, although more knowledge is necessary to verify their effectiveness and acceptability. Further, it is important to evaluate the effects of self-help and low intensity interventions regarding their effects both on psychological and physiological variables.

This study will provide knowledge about the effectiveness and acceptability of two technology-based self-help interventions aimed to induce lifestyle changes among participants who have risk factors for metabolic syndrome together with distress symptoms. Both of these interventions include minimal professional contact. However, the content of the programs and the way they are provided are different. The web-based self-help intervention is based on health check and coaching program, and the mobile-based self-help intervention on acceptance, mindfulness and value-based exercises and materials. The Health Check –program includes comprehensive health assessment tools, and the user receives feedback on one’s life habits and their impact on health. The acceptance and mindfulness –based mobile application contains short exercises that teach acceptance and mindfulness skills to be applied in daily life.

In addition to the two technology-based low intensity interventions, this study increases our understanding of the effectiveness and suitability of acceptance, mindfulness and value-based brief group intervention among participants with obesity and stress problems. There is increasing interest for Acceptance and Mindfulness-based Cognitive Behavior Therapies [76,77]. For example, Gregg, Almada, and Schmidt (2011) have stated that lifestyle interventions applying awareness-focused and value-based interventions might be easier for patients to understand. Thus, this study increases our knowledge of the effectiveness and acceptability of acceptance and mindfulness –based CBT interventions within the area of behavioral medicine.

### Strengths

This study has several strengths. A randomized controlled design was used including several intervention groups with follow-up measurements. Relatively large number of participants from different cities and regions of the country were included in the study. The three interventions are well described including either a protocol or a structured technology intervention. Thus, the interventions were provided in a similar way in different research centers. The study includes exceptionally large number of psychological and physiological measurements. In addition to questionnaires, several behavioral assessments and objective laboratory measurements were used. As a consequence it is possible to investigate wide scope of effects produced by the interventions. This study provides also new knowledge of the psychological change processes associated with low-intensity self-help health interventions and their outcome. We are able to investigate whether the processes of change as reflected by measures of psychological flexibility and mindfulness are similar between the different intervention approaches.

### Limitations

This study has several limitations that need to be observed and discussed. It is possible that large number of measurements and intensive assessment procedures where self-monitoring of participants hardly could be avoided may induce beneficial changes on the control group as well as an additional effect on the active intervention groups. Similarly, because of the exclusion criteria it is probable that participants had relatively mild health problems. Among participants experiencing weight concerns and stress there is probably a large variation of the reasons why they attended to the study as well as their motivation for life style changes. All these issues might limit the detection of the effects of the psychological interventions and possibilities to observe statistical differences between the groups. The fact that the participants were recruited via advertisements in local newspapers increases possibility that the participants included in the study were highly motivated, and the effects of the interventions might be different when used in another context.

## Conclusions

This study increases knowledge of the effects of low-cost brief interventions among obese individuals experiencing stress. The study will provide evidence-based psychological and physiological data on the effects of low intensity self-help technology –based interventions as well as of acceptance, mindfulness and value-based interventions. If successful, information to whom these interventions would be best suitable, and what mechanisms mediate the intervention effects can be obtained.

## Competing interests

The authors have no competing interests to declare. The study has been funded by Tekes (the Finnish Funding Agency for Technology and Innovation). The study is part of the SalWe Research Programme for Mind and Body. SalWe Ltd. is a Strategic Centre for Science, Technology and Innovation in Health and Well-being. In addition to Tekes, Duodecim Medical Publications Ltd provides funding and support for the study. Firstbeat Technologies Ltd and Vivago Ltd provide devices for heart rate variability and actigraphy measurements in the trial.

## Authors’ contributions

RL, MK, ME are the principal investigator of the study, and have participated in the design of the study, coordination and helped to draft the manuscript. ES, TM, LK, JP, EJ, RK, SR, KP, SP, HJ, JL, UMK, participated in planning and constructing the study, they were responsible for organizing and collecting the data, and helped to draft the manuscript. ES and RL constructed the face to face –intervention, and ES was one of the coaches of the face-to-face intervention. RL, ES and TM participated in planning and constructing the mobile intervention. ME, EM, KK, AA, participated in planning and constructing the mobile intervention, and constructing the evaluation user experiences of the mobile and web interventions. OS, EK participated in planning the study and were responsible organizing and providing the web intervention. All authors approved the final version.

## Authors’ information

Miikka Ermes and Marjukka Kolehmainen are equal contributors as a last author.

## Pre-publication history

The pre-publication history for this paper can be accessed here:

http://www.biomedcentral.com/1471-2458/14/310/prepub
